# A Machine Learning Prediction Model of Respiratory Failure Within 48 Hours of Patient Admission for COVID-19: Model Development and Validation

**DOI:** 10.2196/24246

**Published:** 2021-02-10

**Authors:** Siavash Bolourani, Max Brenner, Ping Wang, Thomas McGinn, Jamie S Hirsch, Douglas Barnaby, Theodoros P Zanos, Northwell COVID-19 Research Consortium

**Affiliations:** 1 Feinstein Institutes for Medical Research Northwell Health Manhasset, NY United States; 2 See Acknowledgments

**Keywords:** artificial intelligence, prognostic, model, pandemic, severe acute respiratory syndrome coronavirus 2, modeling, development, validation, COVID-19, machine learning

## Abstract

**Background:**

Predicting early respiratory failure due to COVID-19 can help triage patients to higher levels of care, allocate scarce resources, and reduce morbidity and mortality by appropriately monitoring and treating the patients at greatest risk for deterioration. Given the complexity of COVID-19, machine learning approaches may support clinical decision making for patients with this disease.

**Objective:**

Our objective is to derive a machine learning model that predicts respiratory failure within 48 hours of admission based on data from the emergency department.

**Methods:**

Data were collected from patients with COVID-19 who were admitted to Northwell Health acute care hospitals and were discharged, died, or spent a minimum of 48 hours in the hospital between March 1 and May 11, 2020. Of 11,525 patients, 933 (8.1%) were placed on invasive mechanical ventilation within 48 hours of admission. Variables used by the models included clinical and laboratory data commonly collected in the emergency department. We trained and validated three predictive models (two based on XGBoost and one that used logistic regression) using cross-hospital validation. We compared model performance among all three models as well as an established early warning score (Modified Early Warning Score) using receiver operating characteristic curves, precision-recall curves, and other metrics.

**Results:**

The XGBoost model had the highest mean accuracy (0.919; area under the curve=0.77), outperforming the other two models as well as the Modified Early Warning Score. Important predictor variables included the type of oxygen delivery used in the emergency department, patient age, Emergency Severity Index level, respiratory rate, serum lactate, and demographic characteristics.

**Conclusions:**

The XGBoost model had high predictive accuracy, outperforming other early warning scores. The clinical plausibility and predictive ability of XGBoost suggest that the model could be used to predict 48-hour respiratory failure in admitted patients with COVID-19.

## Introduction

On March 11, 2020, COVID-19, the disease caused by SARS-CoV-2 infection, was declared a pandemic by the World Health Organization [1]. As of December 16, 2020, there were more than 17 million confirmed COVID-19 cases and over 300,000 deaths in the United States. During the first wave, New York was the epicenter of the pandemic in the United States, with over 390,000 cases and 30,000 deaths before the summer [2].

Respiratory failure is the leading cause of death among patients with COVID-19, with up to one-third of patients admitted with COVID-19 requiring invasive mechanical ventilation (IMV) [3-8]. The decision to initiate IMV in these patients is not straightforward. Many patients with severe disease appear comfortable despite profound hypoxemia, and they are commonly managed with supplemental oxygen, self-proning, and close monitoring [9,10]. However, some of these patients subsequently deteriorate and require IMV following transfer from the emergency department (ED). This subgroup has worse outcomes than those placed on IMV initially [11]. Before the surge of COVID-19, patients initially admitted to a non–critical care setting who needed an unplanned transfer to an intensive care unit (ICU) had greater morbidity and mortality than those admitted directly to a critical care unit [12-14]. Thus, accurately identifying patients at high risk for deterioration could improve clinical outcomes as a result of closer monitoring, direct admission to a critical care unit, or earlier discussions regarding patient preferences and goals of care.

Methods of identifying patients at high risk for or in the early stages of clinical deterioration have been actively researched for decades. The field has generated many severity-of-illness calculators, early warning scores, and, more recently, predictive analytic tools that use advanced machine learning and artificial intelligence [15-23]. Our goal was to derive a prediction model that estimates the risk of short-term (<48 hours) respiratory failure for patients with COVID-19 who were not initially placed on IMV. Such a tool could improve outcomes by avoiding delayed admission to a critical care unit, resulting in the provision of additional respiratory support and closer monitoring, or the initiation of earlier discussions around the goals of care.

## Methods

### Overview

This retrospective observational cohort drew data from 13 acute care hospitals of Northwell Health, the largest health care system in New York State. Data were extracted from the electronic health record (EHR) Sunrise Clinical Manager (Allscripts). EHRs were screened for adult patients (aged ≥21 years) who received a positive test result for SARS-CoV-2 based on a nasopharyngeal sample tested using polymerase chain reaction assays. Included patients were hospitalized and were discharged, died, or spent a minimum of 48 hours in the hospital between March 1, 2020, and May 11, 2020. For patients who had multiple qualifying hospital admissions, only the first hospitalization was included. Patients who were transferred between hospitals within the health system were treated as one hospital encounter. A total of 11,919 patients were identified. Patients were excluded if they were placed on mechanical ventilation prior to inpatient admission. A total of 11,525 patients remained for analysis. The Institutional Review Board of Northwell Health approved the study protocol and waived the requirement for informed consent.

### Data Acquisition

Data collected from EHRs included patient demographics, comorbidities, home medications, initial vitals and laboratory values, treatments (eg, oxygen therapy, mechanical ventilation), and clinical outcomes (eg, length of stay, discharge, mortality). Vitals and laboratory testing were restricted to those obtained while the patient was in the ED.

### Outcomes

The target outcome variable was defined as intubation and mechanical ventilation within 48 hours of admission. In the EHR, the admission time was recorded, and the intubation event was defined as the first time mechanical ventilation was recorded.

### Predictive Machine Learning Model

We evaluated three predictive models: XGBoost, XGBoost + SMOTEENN (combined oversampling using SMOTE and undersampling using edited nearest neighbors) [24], and logistic regression [25]. XGBoost combines a recursive gradient–boosting method, called Newton boosting, with a decision-tree model. Given that each tree is boosted in parallel, the model efficiently provides accurate predictions [26]. Furthermore, because each tree is boosted recursively and in parallel, the model benefits from the high interpretability of the variable importance features.

The XGBoost + SMOTEENN method involves combined oversampling using SMOTE and undersampling using edited nearest neighbors on the training set before training an XGBoost model [27]. This method has been shown to have the best performance in the resampling data sets [28]. Furthermore, in our experience, when using any of the oversampling or undersampling methods alone, calibration of the model is severely affected. However, when we combine oversampling the minority class with undersampling of the majority class, we found that we get a more accurate model both in terms of discriminability and minimizing the effect on the calibration of the model.

For every learning framework, we validated the model with external validation using each hospital (ie, for each fold, one hospital was picked as a testing set and the others as a training set). Only hospitals with >1000 patients with COVID-19 in the data set were picked for the testing sets, and a random sample of 1000 patients was picked to be our testing set for each fold. Grid search was used to hypertune the parameters of the respective models. The XGBoost model was tuned based on min_child_weight, gamma, subsample, colsample_bytree, and max_depth parameters, and the ranges of the values were 1-20, 0.5-20, 0.2-1.0, 0.2-1.0, and 2-40, respectively.

When data were missing, we imputed weighted k-nearest neighbors [29] for numerical values and added a category “missing” for categorical values. We used one-hot [30] to encode categorical variables as a one-hot numeric array. The most important variables were calculated based on a decrease in the mean Gini coefficient (ie, the variables most useful in splitting the data to help make a prediction) for XGBoost and XGBoost + SMOTEENN; and by the absolute value of the regression coefficient for logistic regression, and were calculated based on the largest hospital testing set. The resulting receiver operating characteristic (ROC) curves and corresponding accuracy, recall (sensitivity), specificity, geometric mean, and F_β_-score were averaged. For the F_β_-score, the β parameter value was designated as β=4 to capture a higher detriment of false negatives than false positives (ie, if we value recall, β times as much as the precision). For definitions of these measures and how they were calculated, see Multimedia Appendix 1.

Calibration curves (reliability curves) were plotted by dividing the testing sets (for each hospital fold) into 10 bins randomly with an increasing fraction of patients that had respiratory failure in the sample. The fraction positives (patients who had respiratory failure) and their mean corresponding predicted value from the corresponding model were depicted and averaged into 10 bins. The Brier score was calculated for each external hospital fold and the mean Brier score and standard deviation were calculated and depicted in the legend of the calibration curve. For further explanation of these measures and how they were calculated, see Multimedia Appendix 1.

Python 2.6 (Python Software Foundation) was used to implement our machine learning framework. The respective prediction models of XGBoost and logistic regression from the scikit-learn application programming interface (API) in Python were used [31]. GridSearchCV from the scikit-learn API was used to perform the grid search and hypertune the parameters. We used the default imblearn API version of the SMOTEENN [27]. SimpleImputer [32] was used for imputations, which were replaced with a new category, “missing.” KNNImputer [33] was used to impute the missing numerical data [29]. The default value for k=5 was not changed. OneHotEncoder from the sklearn API was used to transform categorical variables to one-hot numeric arrays.

### Modified Early Warning Score

The Modified Early Warning Score (MEWS) was computed from patient vital signs (Multimedia Appendix 2) and is a variant of other known and used risk scores [34,35]. The MEWS ranges from 0 to 15 and incorporates heart rate (beats per minute), respiratory rate (breaths per minute), systolic blood pressure (mm Hg), and body temperature (degrees Celsius). In our data set, one MEWS subcomponent, the AVPU (alert, verbal, pain, unresponsive) neurologic assessment, had a significant amount of missing data (>80%; data not shown) and was not included in the MEWS calculation for this project. An elevated MEWS indicates a risk for clinical instability, including death or the need for ICU admission [36]. In 2012, our health system created a custom modification that was incorporated into the EHR. It includes automatic calculation and display of MEWS and other modules via Arden Syntax Medical Logic Modules [37]. Based on local health system guidelines, any score ≥7 requires an escalation in intensity of care. For example, MEWS >7 requires increased frequency of vital sign measurement (every 2 hours), MEWS >8 requires evaluation by a licensed independent provider, MEWS >9 requires consideration of evaluation by a rapid response team, and MEWS >10 requires a change in the level of service per a defined protocol. For the MEWS, we chose the highest value the patient had while in the ED.

## Results

### Patient Characteristics

During the study period, we identified 11,525 patients admitted from the ED with a diagnosis of COVID-19. Of these, 933 (8.0%) were placed on IMV within 48 hours of admission. Baseline characteristics (demographics, baseline vital signs, and laboratory measurements) for all patients are shown in Table 1, stratified by study outcome. Comorbidities were captured from ICD-10 codes listed in the EHR.

**Table 1:** Demographic, clinical, and laboratory data from hospitalized patients.

Variables		Not intubated (n=10,592)	Intubated (n=933)	Missing (%)
**Demographic characteristics**				
	Age (years), median (IQR)	65.00 (54.00-77.00)	66.00 (56.00-75.00)	0
	Female, n (%)	4530 (42.8)	327 (35.0)	0
	Primary language, English, n (%)	8498 (80.2)	746 (80.0)	0
**Race, n (%)**				0
	Black	2199 (20.8)	236 (25.3)	N/A^a^
	Asian	889 (8.4)	77 (8.3)	N/A
	White	4148 (39.2)	310 (33.2)	N/A
	Declined	71 (0.7)	8 (0.9)	N/A
	Other	2884 (27.2)	268 (28.7)	N/A
	Unknown	401 (3.8)	34 (3.6)	N/A
**Ethnicity, n (%)**				0.1
	Hispanic or Latino	2238 (21.1)	202 (21.7)	N/A
	Not Hispanic or Latino	7685 (72.6)	648 (69.5)	N/A
	Declined	43 (0.4)	1 (0.1)	N/A
	Unknown	618 (5.8)	82 (8.8)	N/A
**Vital signs**				
	Systolic blood pressure (mm Hg), median (IQR)	134.00 (118.00-150.00)	134.00 (115.00-151.75)	0.5
	Diastolic blood pressure (mm Hg), median (IQR)	79.00 (70.50-87.00)	77.00 (69.00-86.00)	0.6
	Heart rate (beats/minute), median (IQR)	94.00 (85.00-102.00)	97.00 (88.50-112.00)	0.4
	Respiratory rate (breaths/minute), median (IQR)	21.00 (18.00-25.00)	24.00 (20.00-32.00)	0.8
	Temperature (°C), mean (SD)	37.77 (0.97)	37.86 (1.11)	1.6
	Oxygen saturation (%), median (IQR)	97.00 (95.00-98.00)	96.00 (93.00-98.00)	1.7
	BMI, mean (SD)	29.12 (7.79)	30.39 (9.21)	47.1
**Laboratory data**				
	White blood cell count (× 10^9^/L), median (IQR)	7.34 (5.45-9.92)	8.25 (6.20-11.50)	9
	Absolute neutrophil count (× 10^9^/L), median (IQR)	5.68 (3.95-8.11)	6.84 (4.76-9.62)	11.5
	Absolute lymphocyte count (× 10^9^/L), median (IQR)	0.90 (0.63-1.27)	0.80 (0.56-1.13)	11.5
	Hemoglobin (g/dL), mean (SD)	12.93 (2.12)	13.14 (2.11)	9
	Platelets (K/μL), mean (SD)	230.17 (101.93)	217.19 (87.45)	9.1
	Sodium (mmol/L), mean (SD),	136.64 (6.21)	135.38 (5.74)	11.9
	Carbon dioxide (mmol/L), mean (SD)	23.61 (3.79)	22.67 (4.68)	11.9
	Creatinine (mg/dL), median (IQR)	1.03 (0.80-1.46)	1.20 (0.92-1.75)	12
	Bilirubin (mg/dL), median (IQR)	0.50 (0.40-0.70)	0.60 (0.40-0.80)	12.5
	Ferritin (ng/mL), mean (SD)	1283.50 (2732.65)	1731.05 (2631.38)	73.2
	Procalcitonin (ng/mL), mean (SD)	1.22 (10.96)	2.12 (8.16)	66.3
	D-dimer (ng/mL), mean (SD)	1871.84 (5306.42)	2659.09 (6798.96)	65.4
	Lactate dehydrogenase (U/L), mean (SD)	455.61 (213.04)	611.05 (272.16)	71
	pH (arterial), mean (SD)	7.42 (0.09)	7.39 (0.11)	96.7
	Partial pressure of oxygen (arterial, mm Hg), mean (SD)	99.90 (65.17)	85.26 (61.42)	94.8
	Partial pressure of carbon dioxide (arterial, mm Hg), mean (SD)	34.66 (9.38)	35.38 (11.45)	94.7
**Comorbidities**				
	Hypertension, n (%)	1183 (11.2)	115 (12.3)	0
	Diabetes, n (%)	685 (6.5)	77 (8.3)	0
	Coronary artery disease, n (%)	148 (1.4)	15 (1.6)	0
	Asthma/chronic obstructive pulmonary disease, n (%)	242 (2.3)	20 (2.1)	0
	Chronic kidney disease, n (%)	99 (0.9)	8 (0.9)	0
	HIV, n (%)	26 (0.2)	1 (0.1)	0

^a^N/A: not applicable.

### Prediction Models for Respiratory Failure

**Figure 1:**
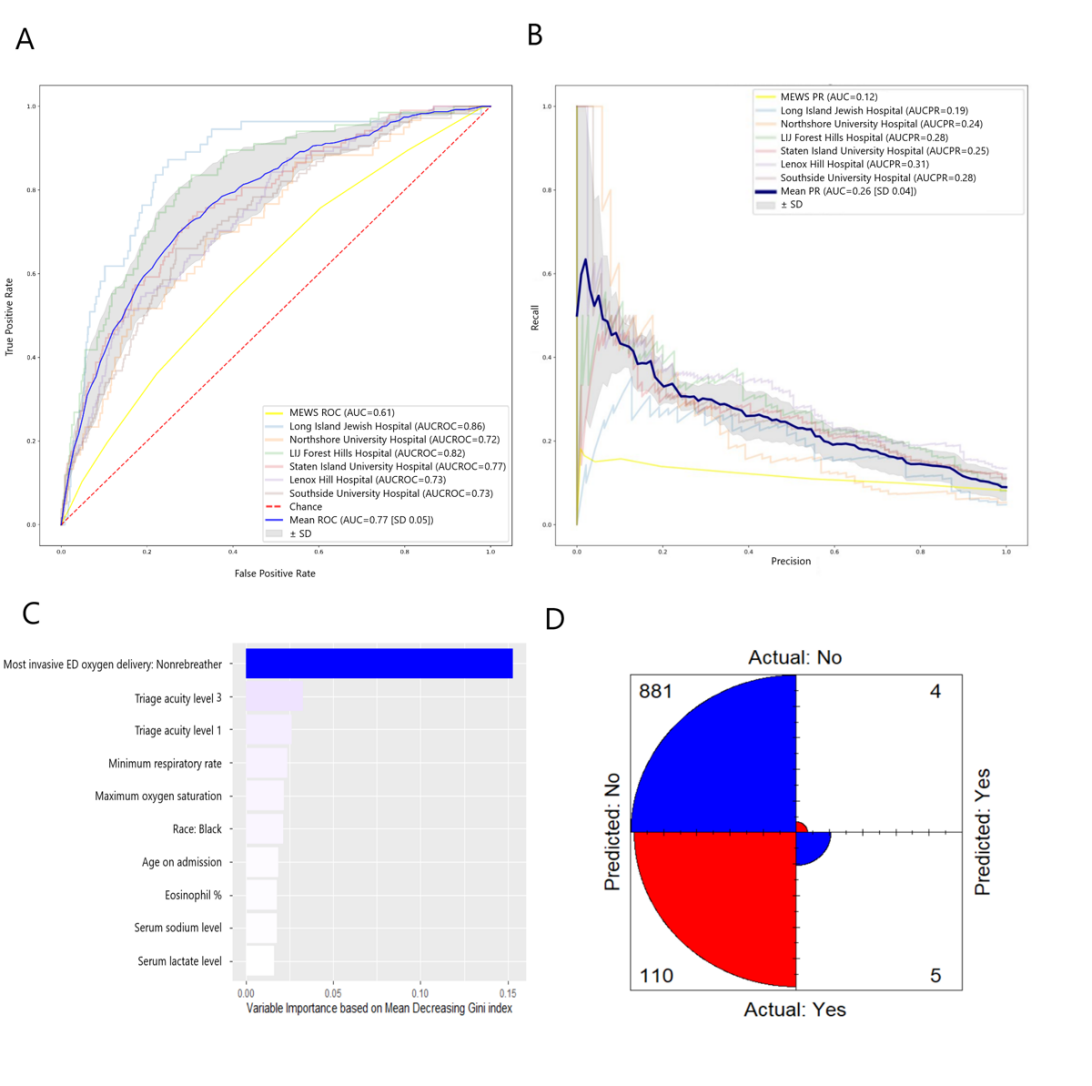
The XGBoost model for predicting respiratory failure within 48 hours. (A) ROC curve and (B) PR curve based on a cross-hospital validation performed by leaving a hospital out as a testing set and using the rest in the training set. Only hospitals with >1000 patients with COVID-19 were selected for testing sets. The mean ROC and PR curves are shown in dark blue and their corresponding standard deviations are shown in gray. The MEWS metrics are shown in light yellow. (C) Measurement of the 10 variables with the highest relative importance based on the amount they reduced the Gini coefficient for the largest hospital testing set. (D) Confusion matrix visually represents the predicted values versus actual prediction for the largest hospital testing set. AUC: area under the curve of ROC; AUCPR: area under the curve of the precision-recall curve; ED: emergency department; LIJ: Long Island Jewish; MEWS: Modified Early Warning Score; PR: precision-recall; ROC: receiver operating characteristic.

Based on XGBoost, the mean area under the curve (AUC) of the ROC (AUCROC) curve was 0.77 (SD 0.05) and the mean AUC of the PR curve (AUCPR) was 0.26 (SD 0.04; Figure 1). The 10 most important variables, in order of decreasing importance, were as follows: most invasive mode of oxygen delivery being a nonrebreather mask, Emergency Severity Index (ESI) values of 1 and 3, maximum respiratory rate, maximum oxygen saturation, Black race, age on admission, eosinophil percentage, serum sodium level, and serum lactate level (Figure 1). The confusion matrix for the model’s largest hospital testing set showed that most false predictions were false negatives (those who were predicted to not require intubation but were intubated within 48 hours). False positives (those who were predicted to require intubation but were not intubated within 48 hours) were the minority of predictions (Figure 1). The model had a mean accuracy of 0.919 (SD 0.028). The corresponding mean precision, recall, specificity, geometric mean, and F_β_-score were 0.521 (SD 0.329), 0.051 (SD 0.030), 0.994 (SD 0.005), 0.337 (SD 0.042), and 0.054 (SD 0.029), respectively (Table 2).

**Figure 2:**
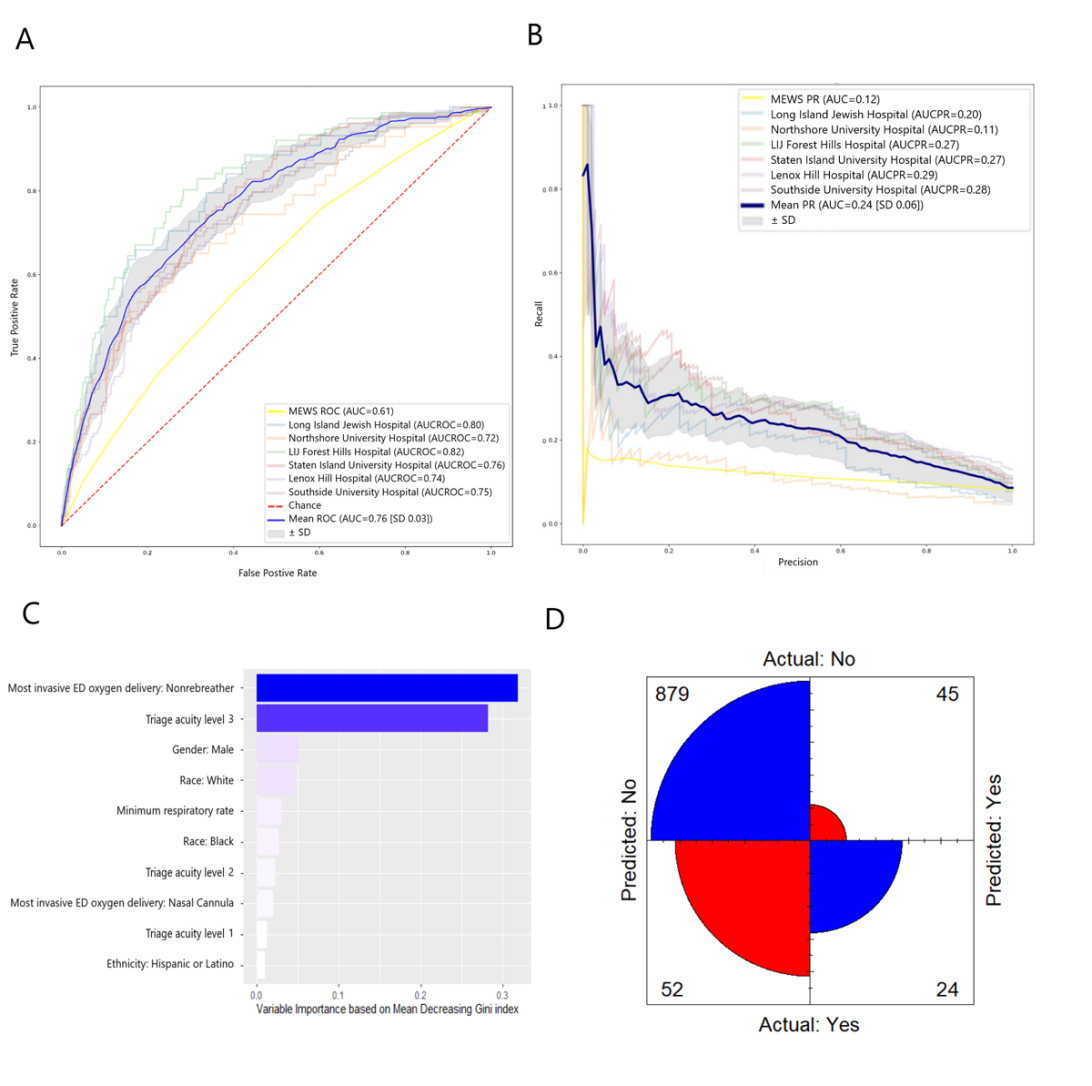
The XGBoost + SMOTEENN model for predicting respiratory failure within 48 hours. (A) ROC curve and (B) PR curve based on a cross-hospital validation performed by leaving one hospital out as a testing set and using the remaining hospitals for the training set. Only hospitals with >1000 patients with COVID-19 were selected for testing sets. The mean ROC and PR curves are shown in dark blue and their corresponding standard deviations are shown in gray. The MEWS metrics are shown in light yellow. (C) The 10 variables with the highest relative importance measured by the amount the variable reduced the Gini coefficient. (D) Mean confusion matrix visually represents the predicted values versus actual prediction. AUC: area under the curve of ROC; AUCPR: area under the curve of the precision-recall curve; ED: emergency department; LIJ: Long Island Jewish; MEWS: Modified Early Warning Score; PR: precision-recall; ROC: receiver operating characteristic.

Based on the XGBoost + SMOTEENN model, the mean AUCs of the ROC and PR curves were 0.76 (SD 0.03) and 0.24 (SD 0.06), respectively (Figure 2). The 10 most important variables, in order of decreasing importance, were as follows: most invasive mode of oxygen delivery being a nonrebreather mask, ESI value of 3, male gender, White race, minimum respiratory rate, Black race, ESI value of 2, most invasive mode of oxygen delivery being nasal cannula, ESI value of 1, and Hispanic ethnicity (Figure 2). The mean confusion matrix showed that most false predictions were false positives (those who were predicted to require intubation but were not intubated within 48 hours). False negatives (those who were predicted to not require intubation but were intubated within 48 hours) were the minority of predictions (Figure 2). Although this model did not have the highest accuracy, it achieved the highest mean recall, geometric mean, and F_β_-score of 0.228 (SD 0.095), 0.508 (SD 0.063), and 0.226 (SD 0.010), respectively. The corresponding mean accuracy, precision, and specificity were 0.893 (SD 0.016), 0.303 (SD 0.089), and 0.955 (SD 0.005), respectively (Table 2).

**Table 2:** Mean area under the curve of the receiver operating characteristic curve, area under the curve of the precision-recall curve, accuracies, precisions, recalls, specificities, geometric means, and Fβ-score (β=4) for models investigated.

Measure	XGBoost, mean (SD)	XGBoost + SMOTEENN, mean (SD)	Logistic regression, mean (SD)	Modified Early Warning Score
Area under the curve of the receiver operating characteristic curve	0.77 (0.05)	0.76 (0.03)	0.70 (0.05)	0.61
Area under the curve of the precision-recall curve	0.26 (0.04)	0.24 (0.06)	0.18 (0.06)	0.12
Accuracy	0.919 (0.028)	0.893 (0.016)	0.915 (0.027)	0.913
Precision	0.521 (0.329)	0.303 (0.089)	0.322 (0.375)	0.165
Recall	0.051 (0.030)	0.228 (0.095)	0.009 (0.013)	0.017
Specificity	0.994 (0.005)	0.955 (0.005)	0.998 (0.002)	0.992
Geometric mean	0.337 (0.042)	0.506 (0.063)	0.285 (0.051)	0.296
F_β_-score	0.054 (0.029)	0.226 (0.088)	0.010(0.014)	0.018

**Figure 3:**
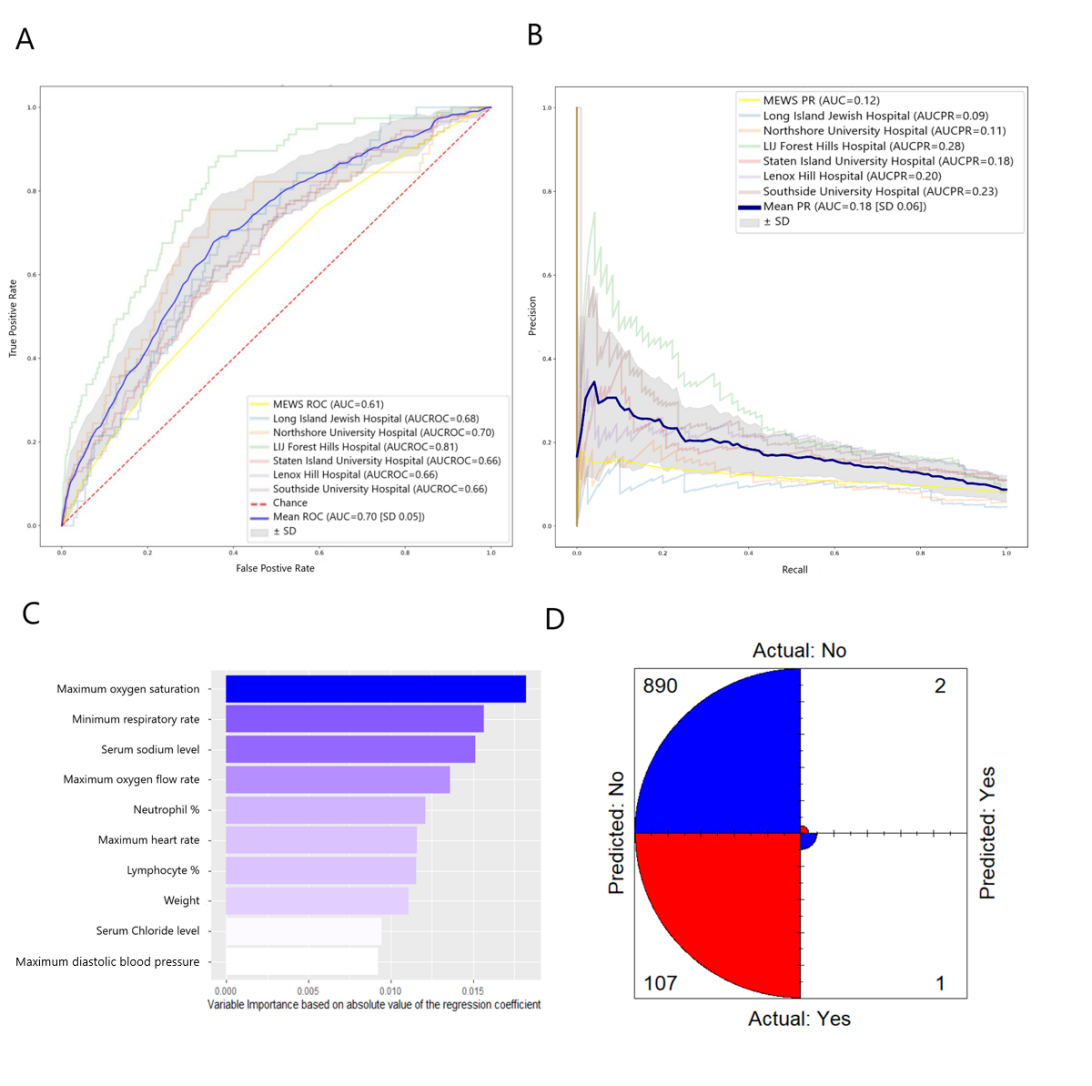
The logistic regression model for predicting respiratory failure within 48 hours. (A) ROC curve and (B) PR curve based on a cross-hospital validation performed by leaving a hospital out as a testing set and using the rest for the training set. Only hospitals with >1000 patients with COVID-19 were selected for testing sets. The mean ROC and PR curves are shown in dark blue and their corresponding standard deviations are shown in gray. The MEWS metrics are shown in light yellow. (C) The 10 variables with the highest relative importance measured by the absolute value of the regression coefficient. (D) Mean confusion matrix visually represents the predicted values versus actual prediction. AUC: area under the curve of ROC; AUCPR: area under the curve of the precision-recall curve; LIJ: Long Island Jewish; MEWS: Modified Early Warning Score; PR: precision-recall; ROC: receiver operating characteristic.

We also examined the performance of a logistic regression model. The mean AUCs of the ROC and PR curves were 0.70 (SD 0.05) and 0.18 (SD 0.06), respectively. Mean accuracy, precision, recall, specificity, geometric mean, and F_β_-score were 0.915 (SD 0.027), 0.322 (SD 0.375), 0.009 (SD 0.013), 0.994 (SD 0.005), 0.285 (SD 0.051), and 0.010 (SD 0.014), respectively (Figure 3 and Table 2). MEWS was used to compare ROC and PR curves. MEWS resulted in AUCs of the ROC and PR curves of 0.61 and 0.12, respectively (Figures 1-3). For MEWS, accuracy, precision, recall, specificity, geometric mean, and F_β_-score were 0.913, 0.165, 0.017, 0.992, 0.296, and 0.018, respectively.

The calibration curves showed that all three models were well calibrated among all hospital folds, although all three deviated from perfect calibration as the fraction of positives increased (Figure 3). The corresponding mean Brier score for XGBoost, XGBoost + SMOTEENN, and logistic regression was 0.071 (SD 0.019), 0.079 (SD 0.016), and 0.077 (SD 0.018), respectively (Figure 3).

## Discussion

We presented three models (two of which were based on XGBoost) for predicting early respiratory failure in patients given a diagnosis of COVID-19 and admitted to the hospital from the ED. One model was tilted toward precision and specificity (XGBoost) and the other was tilted toward recall (XGBoost + SMOTEENN). These models are based on baseline characteristics, ED vital signs, and laboratory measurements. Using an automated tool to estimate the probability of respiratory failure could identify at-risk patients for earlier interventions (eg, closer monitoring, critical care consultation, earlier discussions about goals of care) and improve patient outcomes.

We evaluated three machine learning models: XGBoost, XGBoost + SMOTEENN, and logistic regression [38-40]. XGBoost is widely used due to its high efficiency and predictability, and it has been used to predict health care outcomes in patients with [41,42] and without [43-45] COVID-19. In our study, XGBoost was the most accurate prediction model, with an accuracy of 0.919 (SD 0.028) and precision of 0.521 (SD 0.329; Figure 1), similar to the findings of another study that examined combined outcomes [46]. However, what is different in our model is that it achieves cross-hospital validation. Such accuracy showcases the ability of the model to separate intubations from nonintubations within the 48-hour window of interest. Such a model would be useful for physicians as it more accurately and consistently identifies patients at high risk for intubation.

We also constructed an XGBoost + SMOTEENN model. SMOTEENN was used to improve the sensitivity of our prediction, as our data set was imbalanced (ie, only ~8% of our COVID-19 cohort were intubated), while keeping deviation from accuracy and calibration of the model to a minimum. Compared to XGBoost, the XGBoost + SMOTEENN model had lower accuracy and precision, but greater recall (or sensitivity; 0.228 [SD 0.095]; Figure 2). This higher sensitivity can identify more patients who require IMV, suggesting that this model may be more suitable for broad or automated screening of patients.

We also examined the performance of a logistic regression model to determine whether a compact, linear model could accurately predict patient risk (Figure 3 and Table 2). Model performance was inferior to the XGBoost model. This supports earlier reports that machine learning techniques outperform classic models of logistic regression in their ability to predict many prognostic and health outcomes [47-49]. Finally, we compared the performance of our predictive machine learning models to the widely used MEWS [36]. MEWS was inferior to all three models described above in most of the measures examined.

Using the most important variables for our models, we identified clinically relevant measures that can best inform clinical decision making (Figures 1, 2). The XGBoost model was accurate and precise, as reflected by the low number of false positives of the model predictions (Figure 1). A more sensitive alternative to this model would be the XGBoost + SMOTEENN model, which had fewer false negatives than XGBoost (Figure 2). Both models share important predictors, such as information about the mode of oxygen delivery, triage acuity, demographic information, and respiratory rate. However, XGBoost (the more accurate model with higher precision) adds serum lactate, sodium, and eosinophil percentage to the top 10 most important variables. This indicates that when precision is important, measures such as lactate can rule out the most severe cases by becoming strong predictors. Among hospitals in Northwell Health, certain hospitals such as Long Island Jewish (which is one of the largest in terms of number of patients with COVID-19) had a high drop in their predictive ability when logistic regression was used. When Long Island Jewish was being validated, the 0.86 AUCROC of the XGBoost model dropped to 0.68 for logistic regression. This could partially be due to the nature of the outcome predicted (choice of ventilation from hospital staff), where one would expect different hospitals to possibly exhibit higher variability, not only for patient demographics, but also for hospital staff therapy choices.

Variable importance metrics revealed that the linear logistic regression models use laboratory variables primarily, whereas nonlinear XGBoost-based models prioritize clinical and demographic variables that better capture hospital-specific behavior (eg, oxygen delivery types prior to intubation) and increase the robustness of the model. However, we need to validate whether providing these variables along with the probability of respiratory failure would decrease the rate of identifying at-risk patients. Further prospective studies and randomized clinical trials are needed for this validation.

**Figure 4:**
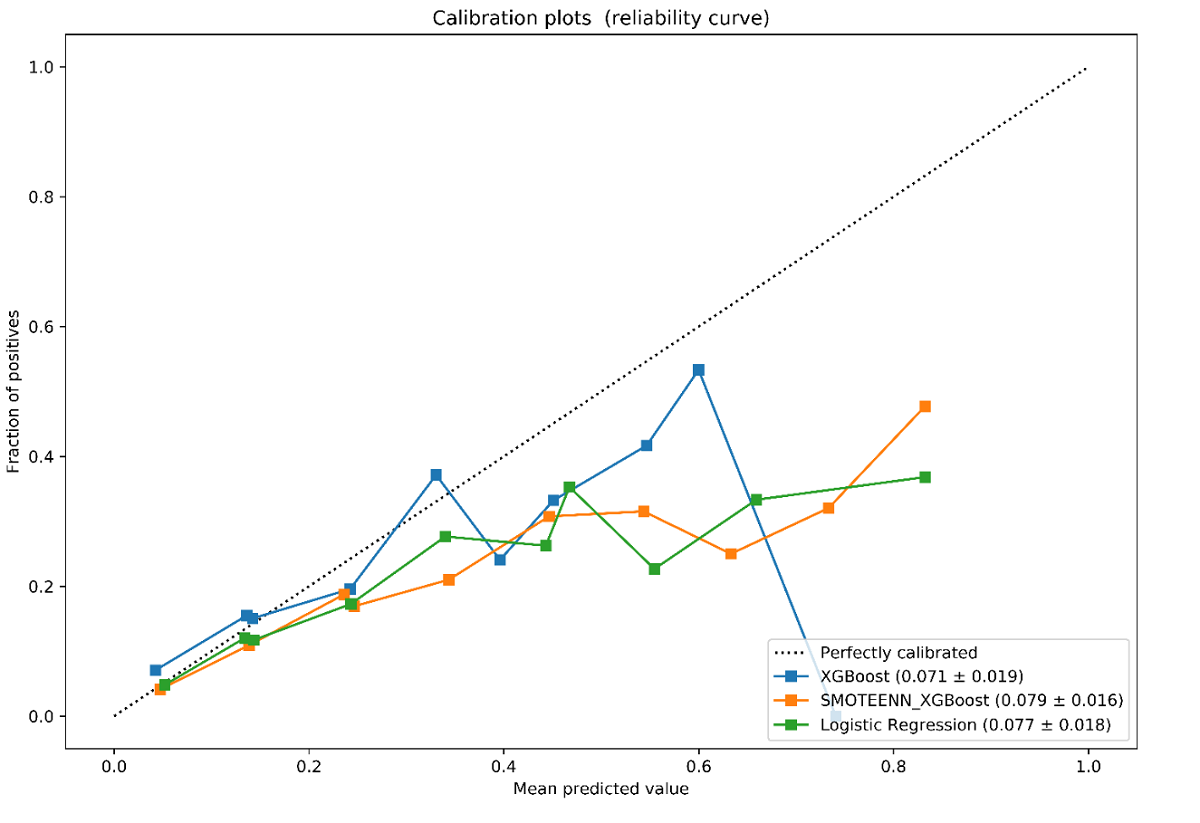
Calibration plots (reliability curve) of the XGBoost, XGBoost + SMOTEENN, and logistic regression models for respiratory failure within 48 hours. Calibration is based on the precision probability (using predict_proba in Python). For creating the plots, sklearn.calibration.CalibratedClassifierCV (in Python) was used by inserting a fraction of positives and mean predicted values into 10 bins with an increasing fraction of positives (respiratory failures) for each hospital fold. The mean Brier score (SD) across all hospitals tested corresponding to the model is shown in the figure legend in parentheses.

When examining the calibration of the models (Figure 4), we found that all models were well calibrated, yet as the fraction of positive cases increased, calibration suffered. This suggests that if a specific population of patients has a greater likelihood of intubation (eg, those aged >70 years, or with specific comorbid conditions), the model would need to be retrained to increase its accuracy and calibration.

Our study has several limitations. We extracted data on intubation timing from our EHR, which may have minor inaccuracies. Although a consistent temporal inaccuracy could create bias in underestimating/overestimating the intubation rate, we believe that these small inaccuracies are overcome by the average calculated from our large number of cases. Another limitation is that we relied on data from a multicenter, single health system for both implementation and validation. Thus, we were unable to externally validate the models in other health systems and hospitals with different protocols, which might affect the model’s performance. In addition, because the study is retrospective, we can only suggest associations and correlations rather than identify the main contributors that lead to intubation and mechanical ventilation. Furthermore, the numerical missing variables were imputed with weighted k-nearest neighbors. Thus, the conclusions made from these variables assume uniformity in patient data based on those missing values. In the case of nonuniformity, the order of variable importance might change. Additionally, some clinical variables included in the model may appear to be obvious correlates of the clinical decision for intubation within 48 hours (eg, having nonrebreather oxygen flow as the most invasive form of ventilation). However, the association of all included variables is not deterministic: only 453 of 2633 patients on nonrebreather oxygen flow in the ED were intubated within 48 hours. In addition, given that these variables are available to clinicians and part of their decision making, we included them in our model. Finally, we used supervised learning on a homogenous database. Although we used cross-hospital validation and retrospectively validated our learning method, external generalizability of these learning methods to other health systems requires validation in prospective studies and randomized trials. Such high-quality evidence could provide more clues on clinical and economic impacts, as well as measures to improve them.

COVID-19 has evolved into an extremely challenging clinical and public emergency worldwide, especially in the New York City metropolitan area. As public health measures attempt to mitigate this disaster by slowing the spread and alleviating the heavy burden placed on health care systems, clinicians must make important decisions quickly and hospital administrators must manage resources and personnel. Furthermore, as predicted by many models [50-52], we are in the midst of a second wave of infection. Our models could inform clinical care by offering complementary performance characteristics (one model with superior recall, the other with greater precision) and supporting clinical decision making as we tackle this unprecedented public health crisis.

## Appendix

Multimedia Appendix 1 Definitions of accuracy, precision, recall, specificity, geometric means, and Fβ-score.

Multimedia Appendix 2 Modified Early Warning Score calculation based on vital sign measurements.

## Abbreviations

AUC: area under the curve
AUCPR: area under the curve of the precision-recall curve
AUCROC: area under the curve of the receiver operating characteristic curve
ED: emergency department
EHR: electronic health record
ESI: Emergency Severity Index
ICU: intensive care unit
IMV: invasive mechanical ventilation
MEWS: Modified Early Warning Score
PR: precision-recall
ROC: receiver operating characteristic
SMOTE: synthetic minority oversampling
SMOTEENN: oversampling using SMOTE and cleaning using edited nearest neighbors
