# Supplementary material for: A Machine Learning Prediction Model of Respiratory Failure Within 48 Hours of Patient Admission for COVID-19: Model Development and Validation
Source: J Med Internet Res. 2021 Feb 10;23(2):e24246. doi: 10.2196/24246 (PMC7879728; doi:10.2196/24246)
Supplement: Multimedia Appendix 1 [file jmir_v23i2e24246_app1.docx]

**Table.** Definitions of Accuracy, Precision, Recall, Specificity, Geometric Means and F_β_-Score

| Term | Definition |
| --- | --- |
| Accuracy | The measure that computes each closeness of the prediction to actual values. In our models, we calculated accuracy using the accuracy_score method of sklearn API in Python. |
| Precision | The ratio of true positives among all the predicted positives (i.e., true positives and false positives). The precision shows the ability of the model to not label an outcome as positive when it is negative. In our models, we calculated precision using the precision_score method of sklearn API in Python. |
| Recall | The ratio of true positives among all the actual positives (i.e., true positives and false negatives). Another name for recall is sensitivity. The recall shows the ability of the model to find all the positives. In our models, we calculated recall using the recall_score method of sklearn API in Python. |
| Specificity | The ratio of true negatives among all the actual negatives (i.e., true negatives and false positives). The specificity shows the ability of the model to find all negatives. In our models, we calculated specificity using the recall_score method of sklearn API in Python and setting pos_label = 0. |
| Geometric mean | The root of the product of sensitivity and specificity. This measure can be used to compare the two models when the classes are imbalanced. Therefore accuracy is not the best measure of comparison among two models. The best value is 1 and the worst value is 0. In our models, we calculated geometric mean using the geometric_mean_score method of imblearn API in Python. |
| F_β_-Score | The weighted harmonic mean of precision and recall. This measure can be used to compare the two models when precision and recall (sensitivity) have different importance, and therefore accuracy is not the best measure of comparison among two models. That is, if we value recall, β times as much the precision. F_β_-Score is often used to compare models when false negatives have higher detriment than false positives and false positives can be tolerated (or vice versa). In our models we calculated this by using the fbeta_score method of sklearn API in python. |
| Calibration curves | Also called reliability diagrams, which shows how reliable the probabilistic predictions are as the fraction of samples belonging to minority class (in our case, those who are to have respiratory failure within 48 hours) increases in the prediction sample. This measure is often useful when in a clinical situation, we would like to know that our prediction will not become unreliable if the patients we are applying it on have a higher or lower fraction overall. For example, in the testing sample 100, if 50 patients are to have respiratory failure,n plots, and by measuring Brier score 6. The calibration is based on how far away from that 50 (how unreliable) is our predictive model when it is predicting that sample. If the prediction model gives us exactly 50%, then the model is perfectly calibrated at that point. This measure can be seen in calibration probability (using predict_proba of python). For creating the plots, we used sklearn.calibration.CalibratedClassifierCV of python which does cross validation as well. |
| Brier Score | This score measures the accuracy of a probabilistic forecast to see how well the model is calibrated. It is measured by the mean score of error in the probability of prediction. Brier score of 0 implies that the model is perfectly calibrated in every tested sample (no error). The further away from 0 the number gets, the more error in calibration of the model. We used python’s sklearn.metrics.brier_score_loss to calculate this metric. |

# 
