# Supplementary material for: A Machine Learning Prediction Model of Respiratory Failure Within 48 Hours of Patient Admission for COVID-19: Model Development and Validation
Source: J Med Internet Res. 2021 Feb 10;23(2):e24246. doi: 10.2196/24246 (PMC7879728; doi:10.2196/24246)
Supplement: Multimedia Appendix 2 [file jmir_v23i2e24246_app2.docx]

**Table**. Modified Early Warning Score calculation based on vital sign measurements*

|  | 3 | 2 | 1 | 0 | 1 | 2 | 3 |
| --- | --- | --- | --- | --- | --- | --- | --- |
| Systolic blood pressure, mmHg | <70 | 70–80 | 81–100 | 101–199 | — | ≥200 | — |
| Heart rate, bpm | — | <40 | 41–50 | 51–100 | 101–110 | 110–129 | ≥130 |
| Respiratory Rate, bpm | — | <9 | — | 9–14 | 15–20 | 21–29 | ≥30 |
| Temperature, ºC | — | <35 | — | 35–38.4 | — | ≥38.5 | — |
| BMI, kg/m2 | — | — | <18.5 | — | 25.1–34.9 | ≥35 | — |
| Age, y | — | — | — | — | 65–74 | 75–84 | ≥85 |

Definition of abbreviations: BMI = body mass index; bpm = beats per minute.

*Each item is scored, and a final score is tallied (range 0 to 15) such that higher values indicate greater risk of clinical decompensation.
